# The pathological significance and potential mechanism of ACLY in cholangiocarcinoma

**DOI:** 10.3389/fimmu.2024.1477267

**Published:** 2024-09-27

**Authors:** Xiaoyan Sun, Xiaofang Zhao, Senyan Wang, Qi Liu, Wenjuan Wei, Jing Xu, Hongyang Wang, Wen Yang

**Affiliations:** ^1^ Translational Medicine Centre, The First Affiliated Hospital of Zhengzhou University, Zhengzhou, Henan, China; ^2^ International Co-operation Laboratory on Signal Transduction, Eastern Hepatobiliary Surgery Institute/Hospital, Naval Medical University (Second Military Medical University), Shanghai, China; ^3^ National Center for Liver Cancer, Naval Medical University (Second Military Medical University), Shanghai, China

**Keywords:** ACLY, cholangiocarcinoma, fatty acid metabolism, ferroptosis, immune microenvironment

## Abstract

**Background and aim:**

Cholangiocarcinoma (CCA) is a rare cancer, yet its incidence and mortality rates have been steadily increasing globally over the past few decades. Currently, there are no effective targeted treatment strategies available for patients. ACLY (ATP Citrate Lyase), a key enzyme in *de novo* lipogenesis, is aberrantly expressed in several tumors and is associated with malignant progression. However, its role and mechanisms in CCA have not yet been elucidated.

**Methods:**

The expression of ACLY in CCA was assessed using transcriptomic profiles and tissue microarrays. Kaplan-Meier curves were employed to evaluate the prognostic significance of ACLY in CCA. Functional enrichment analysis was used to explore the potential mechanisms of ACLY in CCA. A series of assays were conducted to examine the effects of ACLY on the proliferation and migration of CCA cells. Ferroptosis inducers and inhibitors, along with lipid peroxide probes and MDA assay kits, were utilized to explore the role of ACLY in ferroptosis within CCA. Additionally, lipid-depleted fetal bovine serum and several fatty acids were used to evaluate the impact of fatty acids on ferroptosis induced by ACLY inhibition. Correlation analyses were performed to elucidate the relationship between ACLY and tumor stemness as well as tumor microenvironment.

**Results:**

The expression of ACLY was found to be higher in CCA tissues compared to adjacent normal tissues. Patients with elevated ACLY expression demonstrated poorer overall survival outcomes. ACLY were closed associated with fatty acid metabolism and tumor-initiating cells. Knockdown of ACLY did not significantly impact the proliferation and migration of CCA cells. However, ACLY inhibition led to increased accumulation of lipid peroxides and enhanced sensitivity of CCA cells to ferroptosis inducers. Polyunsaturated fatty acids were observed to inhibit the proliferation of ACLY-knockdown cells; nonetheless, this inhibitory effect was diminished when the cells were cultured in medium supplemented with lipid-depleted fetal bovine serum. Additionally, ACLY expression was negatively correlated with immune cell infiltration and immune scores in CCA.

**Conclusion:**

ACLY promotes ferroptosis by disrupting the balance of saturated and unsaturated fatty acids. ACLY may therefore serve as a potential diagnostic and therapeutic target for CCA.

## 1 Introduction

CCA is a malignant tumor originating from biliary epithelial cells. It can be categorized into intrahepatic cholangiocarcinoma, perihilar cholangiocarcinoma and extrahepatic cholangiocarcinoma according to anatomical location in the bile duct tree (1, 2). CCA is a rare but high aggressive cancer. The 5-year relative survival rates range from 2% to 15% for intrahepatic cholangiocarcinoma (ICC) and 2%-30% for extrahepatic cholangiocarcinoma (ECC) (3). Surgical resection may be a viable option for early-stage CCA. Unfortunately, most CCA patients are diagnosed at advanced stages, for which severely limited therapeutic options are available (4, 5).Therefore, there is a great clinical need to identify new prognostic biomarkers and therapeutic targets for CCA.

Metabolic reprograming is a hallmark of cancer. Enhanced lipid synthesis is among the most prominent metabolic characteristics of cancer (6). Most normal cells and tissues preferentially utilize circulating lipids, whereas cancer cells predominantly rely on the *de novo* fatty acid synthesis pathway to enhance membrane lipid saturation and helps cancer cells to survive both carcinogenic and therapeutic challenges (7, 8).ACLY is a pivotal enzyme at the crossroads of glucose and lipid metabolism. Cytosolic citrate is broken down by ACLY to oxaloacetate and acetyl-CoA (9). Acetyl-CoA is required for lipogenesis and cholesterogenesis. Besides, acetyl-CoA is involved in the acetylation of proteins (10, 11). Thus, ACLY is a key enzyme in cancer metabolism. Previous studies have proved that the expressions of ACLY were increased in gastric cancer, ovarian cancer, prostate cancer, lung cancer, etc., and correlated with increased tumor aggressiveness and poor patient prognosis (12–14). We had also recently demonstrated that ACLY overexpression or activation drive hepatocellular carcinoma stemness and metastasis (15). In addition, it was reported that ACLY play crucial role in breast cancer stemness and metastasis (16). So, it was believed that ACLY promote tumor progression by enhancing various cancer-associated processes, including stemness, metastasis, and metabolic reprogramming.

Ferroptosis, an iron-dependent form of programmed cell death, is characterized by an overproduction of lipid peroxidation and the accumulation of reactive oxygen species (ROS) (17). Recently, ferroptosis has garnered significant attention as a promising target in anticancer therapies. The process of fatty acid metabolism is closely tied to ferroptosis. An increase in PUFA levels or a disruption in their regulatory mechanisms can lead to their peroxidation, thereby inducing ferroptosis. On the other hand, monounsaturated fatty acids (MUFAs), due to their stability, can mitigate lipid peroxidation by replacing PUFAs (18, 19). As such, in the context of cancer treatment, strategies that enhance PUFA levels, activate the PUFA regulatory system, or reduce MUFA synthesis could potentiate ferroptosis, thus improving therapeutic efficacy. *De novo* lipogenesis (DNL), the process by which excess carbohydrates and proteins are converted into saturated and monounsaturated fatty acids (SFAs and MUFAs), is preferentially utilized by tumor cells to mitigate the risk associated with excessive intake of unsaturated fatty acids (20). ATP citrate lyase (ACLY), a cytosolic enzyme, plays a crucial role in DNL by converting citrate and coenzyme A (CoA) into acetyl-CoA, which serves as a precursor for lipid synthesis (21). Given this context, we propose that inhibiting ACLY might enhance ferroptosis in cholangiocarcinoma (CCA) cells, offering a potential avenue for therapeutic intervention.

In the current study, we systematically investigated the expression, prognostic value, immune infiltration, co-expressed gene network and function analysis in CCA. We discovered that the expressions of ACLY was upregulated in CCA compared with adjacent tissue and correlated with poor patient prognosis. ACLY inhibition facilitated ferroptosis by increasing polyunsaturated fatty acids (PUFAs) uptake. In addition, our analyses revealed that ACLY expression levels are negatively correlated with cancer stemness, immune infiltration, and the efficacy of immunotherapy in CCA. Therefore, ACLY could serve as a new potential therapeutic and prognostic biomarker for CCA.

## 2 Methods and materials

### 2.1 Patients and specimens

Tissue microarrays composed of tumor samples and normal tissue were obtained from 87 patients who pathologically diagnosed as CCA in the Eastern Hepatobiliary Surgery Hospital (Shanghai, China) from February 2018 to December 2019. The use of patient information and tissues were approved by the Ethics Committee of the Second Military Medical University. Informed consent was obtained from patients prior to surgical resection.

### 2.2 Data collection and preprocessing

Transcriptional and proteomic expression data, along with corresponding clinical characteristics of cholangiocarcinoma (CCA) patients, were sourced from several databases. Fan-CCA dataset was downloaded from biosino NODE database (NODE database: OEP001105, https://www.biosino.org/node/project/detail/OEP001105) (22). Four gene expression profiles were obtained from GEO database (https://www.ncbi.nlm.nih.gov/geo/): GSE107493 (including 30 CCA samples and 27 adjacent nontumor samples), GSE3225 (including 149 CCA samples and 6 adjacent nontumor samples), GSE89749 and GSE244807. E-MTAB-6389 dataset was downloaded from EMBL-EBI database (https://www.ebi.ac.uk/biostudies/arrayexpress/studies/E-MTAB-6389). For pan-cancer analysis, normalized RNA-seq data for 33 tumor types were obtained from The Cancer Genome Atlas (TCGA).

### 2.3 Single‐gene differential analysis and functional enrichment analysis of ACLY in CCA

The “limma” R package was used to screen differential expression genes. Genes with p <0.05 and absolute log2fold change >=0.8 were considered as differentially expressed. These differentially expressed genes were entered into the STRING database, and protein–protein interaction (PPI) of differentially expressed genes was determined using Cytoscape software network analysis. Then, the hub genes were identified using the MCODE plugin. Gene Ontology (GO) terms and Kyoto Encyclopedia of Genes and Genomes (KEGG) pathways were used to discover the functional roles of differentially expressed genes. A gene set was considered enriched if the statistical significance of its enrichment score, as indicated by the false discovery rate (FDR), was less than 0.05. All enrichment analysis were performed by R clusterProfiler (v3.14.3) package. The stemness index was calculated using the single-sample gene set enrichment analysis (ssgsea) algorithm.

### 2.4 Immunoinfiltration analysis of ACLY

The enrichment score of immune cell infiltration was calculated by ‘XCELL’. Correlation analysis of ACLY with immune cell was carried out by R packages ‘corrplot’. ESTIMATE algorithms were used to generate immune score, stromal score and ESTIMATE score.

### 2.5 Immunohistochemistry staining evaluation

The tissue microarray chips were incubated at 60°C for 1h, then dewaxed in xylene and hydrated in gradient alcohol. Block endogenous peroxidase activity by incubating sections in 3% H_2_O_2_ solution in methanol. Antigen retrieval was performed by heating slide in citrate buffer. Block buffer was used to block nonspecific antigens. Tissue sections were incubated with polyclonal rabbit anti-ACLY (dilution 1:300, abcam, ab235926) overnight at 4°C. The slide was incubated with an HRP-labeled goat anti-rabbit secondary antibody 30min at 37°C. Then, the samples were stained with diaminobenzidine (MXB, MAX-001), counterstained with hematoxylin, and dehydrated with gradient alcohol. Immunohistochemistry (IHC) staining scores were calculated based on both staining intensity and staining extent.

### 2.6 Cell culture and transfection

The CCA cell lines HUCCT1 and RBE were obtained from Shanghai Cell Bank of the Chinese Academy of Sciences, China. These cell lines were cultured in RPMI 1640 medium supplemented with 10% Fetal Bovine Serum (FBS), penicillin (100 IU/ml) (Gibco), and streptomycin (100 μg/ml) (Gibco). The lentivirus-mediated shRNA expressing vector targeting human ACLY (target sequence: 5′-CCACAGCTAGAACTTATCAAA-3′) were purchased from Shanghai Genechem. For lentivirus infection, viral solutions were added to cell culture medium containing polybrene (4 μg/ml); 48 hours after infection, cells were selected using puromycin (2 μg/ml) and tested for ACLY expression by quantitative reverse transcription PCR (qRT-PCR) or immunoblotting.

### 2.7 Cell viability assay

For cell proliferation assay, RBE and HUCCT1 cells were seed into 96-well plate (1×10^3^ cell per well). At indicated time points, fresh medium with 10% CCK8 solution (A311-01, Vazyme) was added to each well and absorbance were measured at 450nm after 1 hours. The cell cytotoxicity assay, 3-4×10^3^ cells per well were plated in 96 well plate, the cell was treated with different dose of compounds and cultured for 48h. Absorbance at 450nm was measured 1 hour after 10% CCK-8 incubation. Each assay was repeated at least three times.

### 2.8 ROS, MDA and lipid peroxidation assay

Cells were plated in 6-well plated and allow to attach overnight. Cells were detached into single cell suspension. Next, highly sensitive DCFH-DA (DOJINDO, Japan) or BODIPY 581/591 C11 working solutions (DOJINDO, Japan) were added to cell and incubated at 37°C for 30 min. After washing with Hanks’ Balanced Salt Solution (HBSS), cells were suspended in 200 μl PBS and fluorescence intensity were detected by flow cytometry (BD, USA); Data were analyzed by the Flow Jo V10 software. For the measurement of intracellular Malondialdehyde (MDA) levels, MDA concentration was measured using an assay kit (M496, DOJINDO, Japan) according to the manufacturer’s instructions.

### 2.9 Statistical analysis

Statistical analyses were performed with GraphPad Prism 6.0 software. The log-rank test was used to compare the risk of death between the two groups. The Kolmogorov–Smirnov test was used to test for normal distribution of the data. If the samples were normally distributed, we used unpaired or paired two-tailed Student’s t-tests to determine the statistical difference between two groups. Statistical significance was given by *P < 0.05, **P < 0.01 and ***P < 0.001; N.S., not significant.

## 3 Results

### 3.1 Elevated expression of ACLY in CCA and other cancers

**Figure 1:**
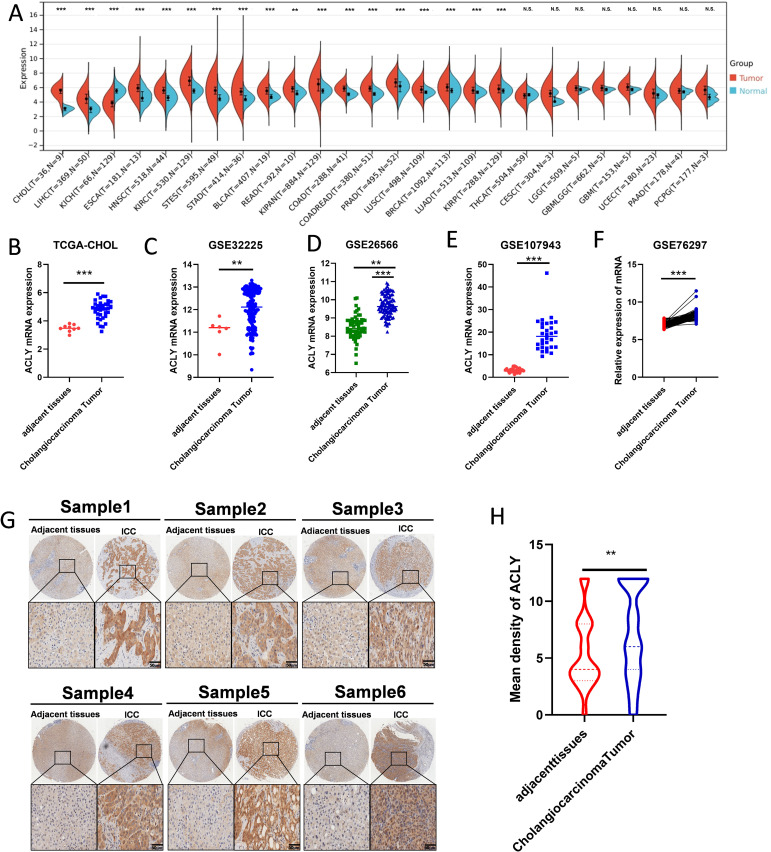
The expression of ACLY was upregulated in CCA and other cancers. **(A)** The expression of ACLY between normal and tumor tissue in pan-cancer. **(B-F)** The mRNA expression of ACLY in CCA tissues compared with their paired adjacent non-tumor tissues in TCGA-CHOL, GSE3225, GSE26556, GSE107943 and GSE76297 datasets. **(G, H)** Immunohistochemistry detection of ACLY protein levels in CCA tissues and the adjacent non-tumor tissues in tissue microarrays (TMAs), represent staining **(G)** and the statistical analysis **(H)**. **P < 0.01 and ***P < 0.001; N.S., not significant.

We examined the expression of ACLY in normal and tumor tissues across a range of cancers. The results revealed that ACLY expression was broadly elevated in several cancers, including CCA, hepatocellular carcinoma, esophageal carcinoma, and head and neck squamous cell carcinoma (**Figure 1A**). Among these, the difference was most significant in CCA. We further validated the elevated expression of ACLY in CCA tissues using multiple datasets, including TCGA-CHOL, GSE3225, GSE26556, GSE107943, and GSE76297 (**Figures 1B-F**). Immunohistochemistry staining demonstrated that ACLY protein levels were higher in CCA tissues compared to adjacent normal tissues (**Figures 1G, H**). Collectively, these data indicate that ACLY expression is significantly upregulated in CCA.

### 3.2 Prognosis value of ACLY in cholangiocarcinoma

**Figure 2:**
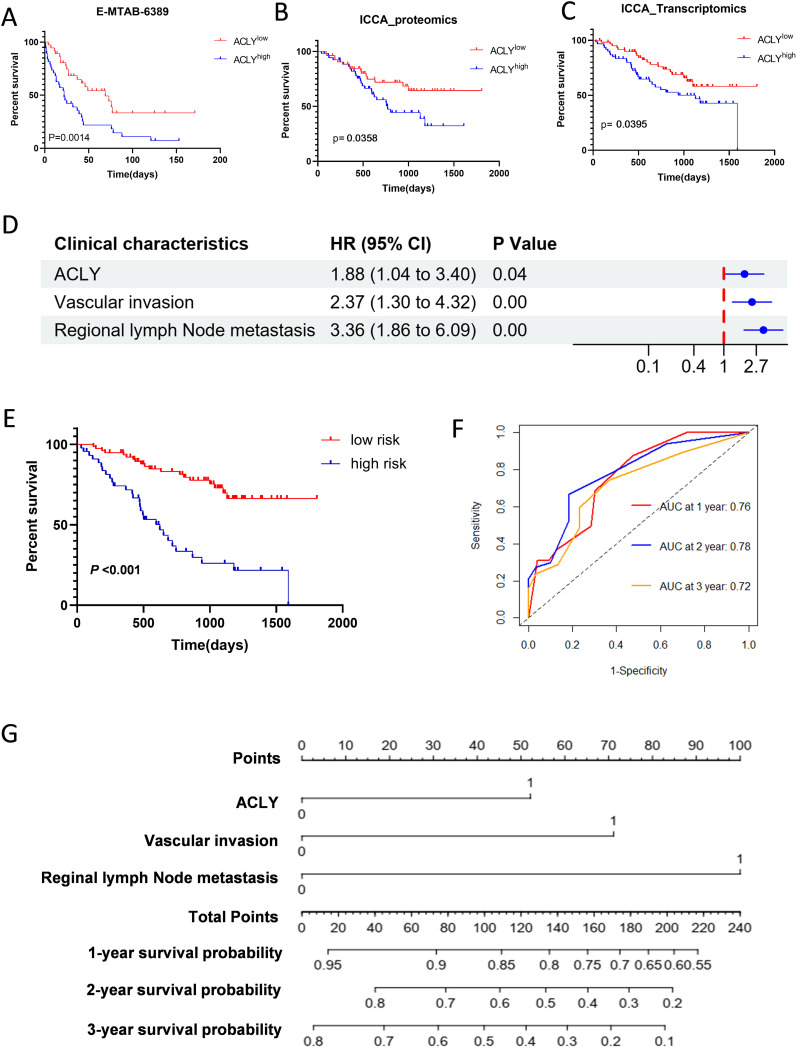
Increased ACLY expression associated with poor overall survival in patients with CCA. **(A-C)** Kaplan-Meier survival analysis of ACLY expression and Overall Survival (OS) in CCA patients from E-MTAB-6398 **(A)** and fan-CCA cohort **(B, C)**. ACLY low expression (red curve), ACLY high expression (blue cure). **(D)** Forest plot showing results of multivariate Cox regression model for exploring potential risk factors for overall survival. **(E)** Based on the multivariate Cox regression model, patients were stratified into low risk and high risk group, Kaplan–Meier method was used to evaluate the difference in the survival status of both risk groups. **(F)** The receiver operating characteristic (ROC) curve was used to evaluate the predictive capacity of the model. **(G)** A nomogram is used to predict survival probability of CCA patients.

To investigate the correlation between ACLY expression and prognosis in CCA, patients in the fan-CCA and E-MTAB-6389 datasets were divided into low and high ACLY expression groups based on ACLY mRNA or protein levels. Kaplan-Meier analysis revealed that patients with high ACLY expression exhibited poorer overall survival (OS) in both datasets (**Figures 2A–C**). In multivariate Cox regression analysis, high ACLY expression was associated with shorter OS (adjusted HR 1.50, 95% CI: 1.110–3.848; P = 0.024) after adjusting for clinical factors such as sex, age, intrahepatic metastasis, HBV infection, vascular invasion, TNM stage, adjuvant treatment, and distant metastasis (**Figure 2D**). Subgroup analyses further indicated that the high ACLY expression group had worse overall survival among patients who were older (P=0.0019; **Supplementary Figures S1A, B**), HBV positive (P=0.0191; **Supplementary Figures S1C, D**), had vascular invasion (P=0.0117; **Supplementary Figures S2E, F**), or received adjuvant treatment (P=0.0190; **Supplementary Figures S2G, H**). Multivariate Cox regression was used to construct clinical prediction model, and results showed that ACLY expression (HR 1.88, 95% CI 1.04-3.40; P=0.04), Vascular invasion (HR 2.37, 95%CI 1.30-4.32; P=0.00) and regional lymph node metastasis (HR 3.36, 95% CI: 1.86-6.09; P=0.00) were independent risk factors (**Figure 2D**). Based on the clinical prediction model, patients were stratified into low risk and high-risk group, Kaplan–Meier analysis showed that patients with high-risk sore had poorer overall survival (OS) (**Figure 2E**). The AUC values for 1-,2-, and 3-year overall survival were 0.76, 0.78, and 0.72 (**Figure 2F**). A nomogram based on the selected prognostic factors was developed for the prediction of CCA patient OS at 1, 2 and 3 years (**Figure 2G**). The nomogram demonstrated that reginal lymph node metastasis contributed the most to prognosis, followed by Vascular invasion and ACLY expression.

### 3.3 Functional enrichment analysis of ACLY in cholangiocarcinoma

**Figure 3:**
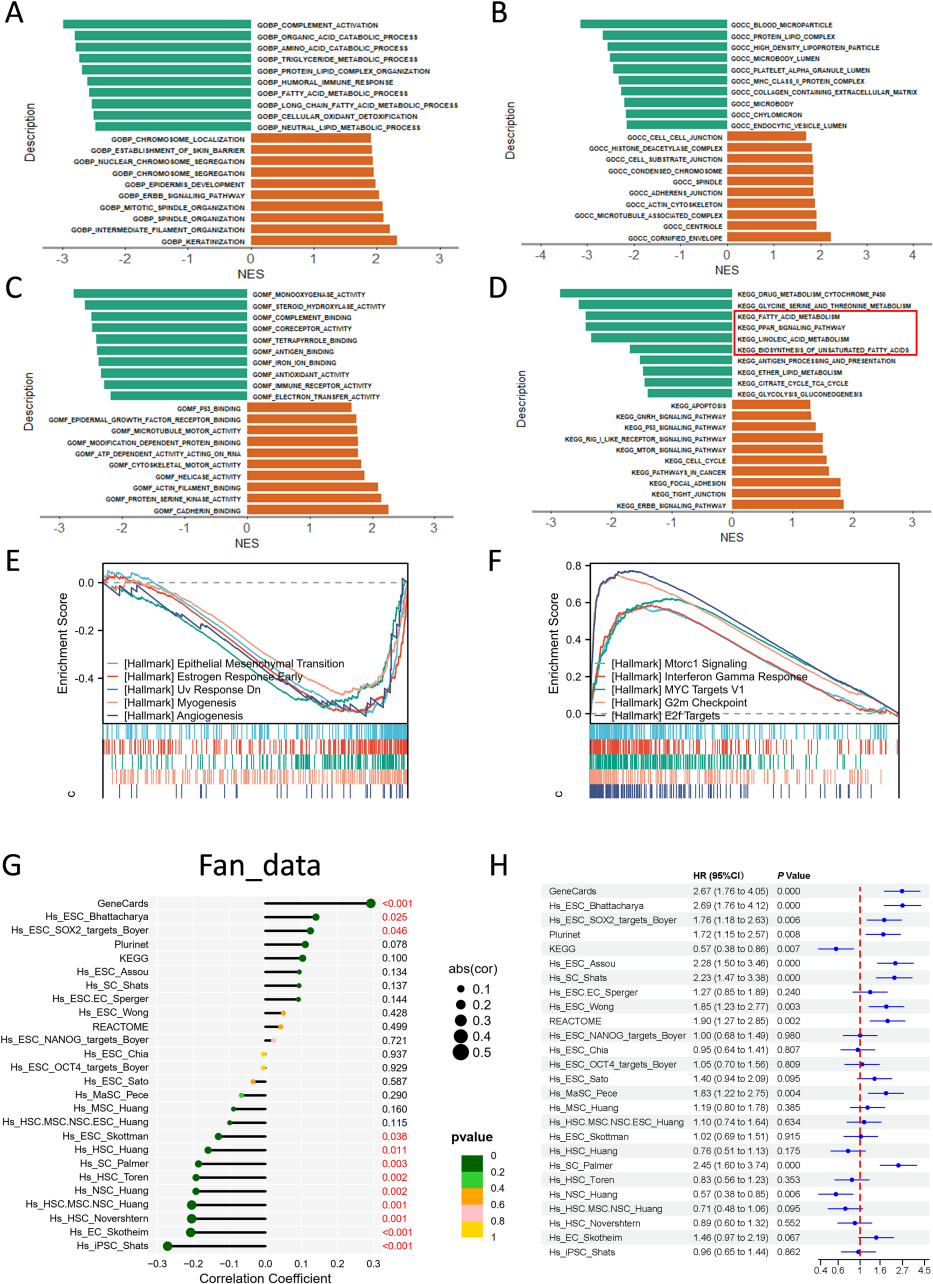
Functional enrichment analysis of ACLY in cholangiocarcinoma. **(A-D)** Results of Gene Ontology (GO) and Kyoto Encyclopedia of Genes and Genomes (KEGG) Analysis of Differentially Expressed Genes in Single Gene Differential Analysis. A Positive Normalized Enrichment Score (NES) Indicates a Positive Correlation Between ACLY Expression and the Terms, whereas a Negative NES Indicates a Negative Correlation. **(E, F)** Gene Set Enrichment Analysis (GSEA) of differentially expressed genes identified in single-gene differential analysis, focusing on hallmark pathways. A Positive Normalized Enrichment Score (NES) Indicates a Positive Correlation Between ACLY Expression and the pathway, whereas a Negative NES Indicates a Negative Correlation. **(G)** Results of the correlation between the expression of ACLY and stemness scores. **(H)** Forest plot showing correlation of different stemness signatures with overall survival.

Single gene differential expression analysis of ACLY was performed using the fan-CCA dataset, and the results are presented in **Supplementary Figure S2A**. A total of 208 genes met the threshold criteria of |log2(FC)| > 0.8 and p value < 0.05. Among these, 58 genes were upregulated, and 151 genes were downregulated. We then constructed a protein-protein interaction (PPI) network using these 208 differentially expressed genes, as shown in **Supplementary Figure S2B**. In the interaction network graph, genes closer to the center exhibited more connections with other genes. Using the MCODE plugin, we identified 15 hub genes: APOH, APOC3, APOB, APOA2, APOA1, AMBP, ALB, AHSG, TTR, SERPINC1, HRG, HPX, FGG, FGA, and F2. The PPI network of these hub genes is illustrated in **Supplementary Figure S2C**. Additionally, a gene co-expression heatmap with ACLY was generated and is shown in **Supplementary Figure S2D**. Gene Ontology (GO), Kyoto Encyclopedia of Genes and Genomes (KEGG), and Gene Set Enrichment Analysis (GSEA) were conducted using the results of single-gene differential expression analysis. The biological processes primarily included complement activation, long-chain fatty acid metabolism, the ERBB2 signaling pathway, and others (**Figure 3A**). The cellular components mainly included blood microparticles, protein-lipid complexes, histone deacetylase complexes, and others (**Figure 3B**). The molecular functions mainly included monooxygenase activity, iron ion binding, antioxidant activity, and p53 binding, among others (**Figure 3C**). **Figure 3D** displays the results of KEGG analysis, which indicated that ACLY was negatively correlated with the biosynthesis of unsaturated fatty acids, the PPAR signaling pathway, and the citrate cycle (TCA cycle), while being positively correlated with the p53 signaling pathway, the cell cycle pathway, and focal adhesion. Gene Set Enrichment Analysis (GSEA) of hallmark gene sets was conducted to Utilizing Gene Set Enrichment Analysis (GSEA), we delineated the functional enrichment of genes in cohorts with high and low ACLY expression. High ACLY expression was associated with epithelial-mesenchymal transition, early estrogen response, angiogenesis, and myogenesis, while low ACLY expression was associated with mTORC1 signaling, interferon-gamma response, MYC targets, G2M checkpoint, and E2F targets (**Figures 3E, F**). Stemness analysis indicated that ACLY exhibited a strong positive correlation with the GeneCard signature, and a high GeneCard stemness score was associated with poor prognosis (**Figures 3G, H**). We also observed similar findings in other CCA cohorts (**Supplementary Figure S2E-H**). ACLY exhibited a positive correlation with most stemness signatures, and certain stemness signature scores were associated with overall survival. These findings underscore the close relationship between ACLY and cellular metabolism, as well as malignant cellular behavior.

### 3.4 ACLY inhibition enhances the sensitivity of cholangiocarcinoma cells to PUFAs

**Figure 4:**
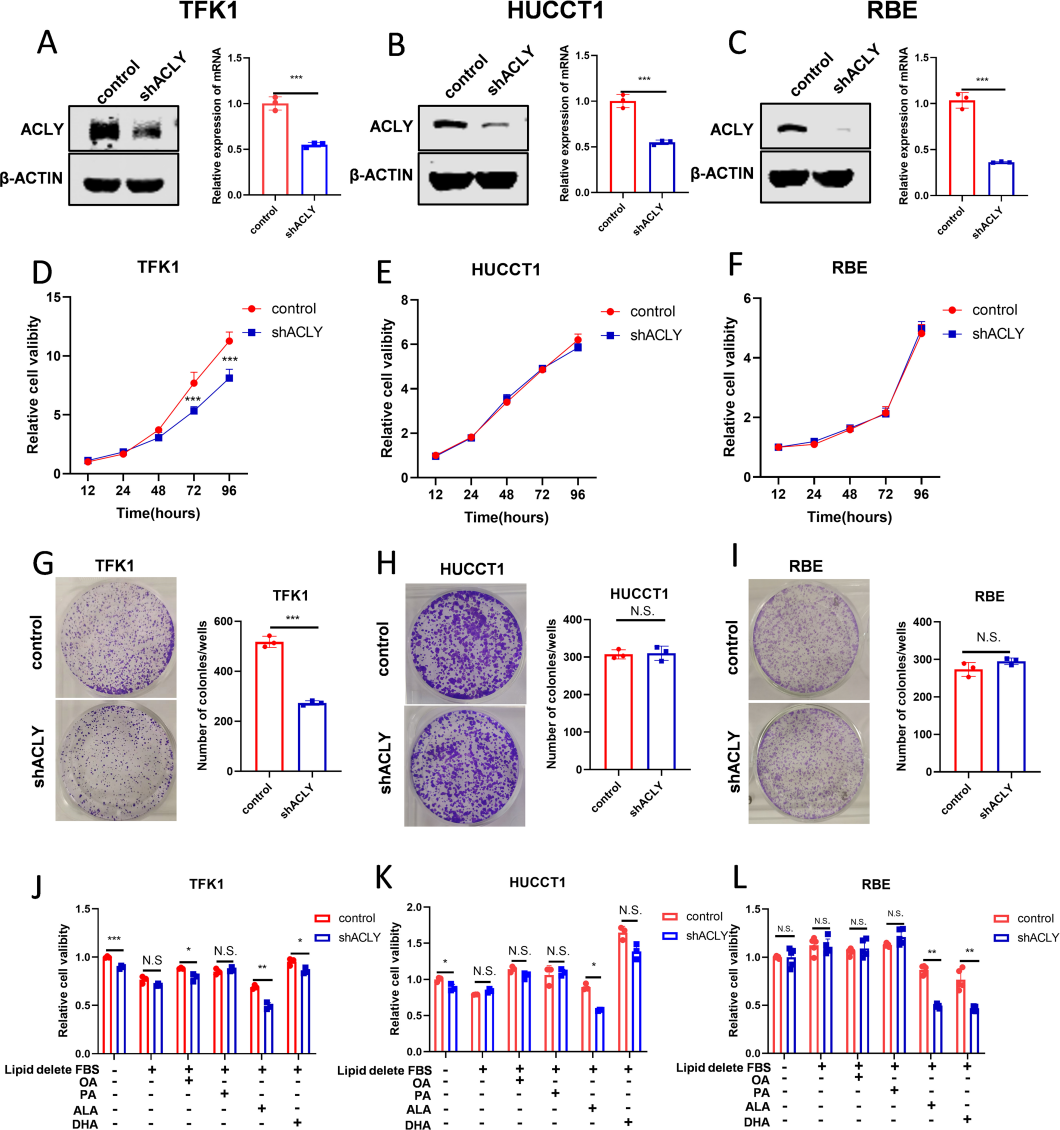
ACLY inhibition enhances the sensitive of cholangiocarcinoma cell to PUFAs. **(A-C)** Western blot and quantitative real-time PCR validate the efficacy of ACLY knockdown in CCA cells. **(D-I)** The effect of ACLY knockdown on cell viability and colony formation abilities of CCA cells. **(J-L)** Impact of fatty acids on cell viability in ACLY control and ACLY knockdown cells. *P < 0.05, **P < 0.01 and ***P < 0.001; N.S., not significant.

Based on clinical evidence and functional analysis, we concluded that ACLY plays a potential role in CCA tumor progression. Western blot analysis examined the expression of ACLY in four CCA cell lines (**Supplementary Figure S3A**), and we selected RBE, HUCCT1, and TFK1 for shRNA-mediated knockdown experiments due to their higher expression levels. The efficacy of ACLY knockdown was validated by qPCR and Western blot (**Figures 4A-C**). Cell viability was measured using CCK8 and colony formation assays. The results showed that knockdown of ACLY did not influence the proliferation of RBE and HUCCT1 cells but suppressed the proliferation of TFK1 cells (**Figures 4D-I**). Additionally, ACLY knockdown had no impact on the migration ability of RBE and HUCCT1 cells but decreased the migration of TFK1 cells (**Supplementary Figures S3B-D**). As ACLY is regarded as the first rate-limiting enzyme involved in *de novo* lipogenesis, we observed the effect of different fatty acids on cell viability. Results indicated that while palmitic acid (PA) and oleic acid (OA) exposure did not significantly inhibit cell growth, polyunsaturated fatty acids (PUFAs) inhibited the growth of ACLY-knockdown cells (**Figures 4J-L**). Therefore, we suggested that ACLY inhibition enhances the sensitivity of CCA cells to PUFAs.

### 3.5 Inhibition of ACLY promotes ferroptosis in CCA cells

**Figure 5:**
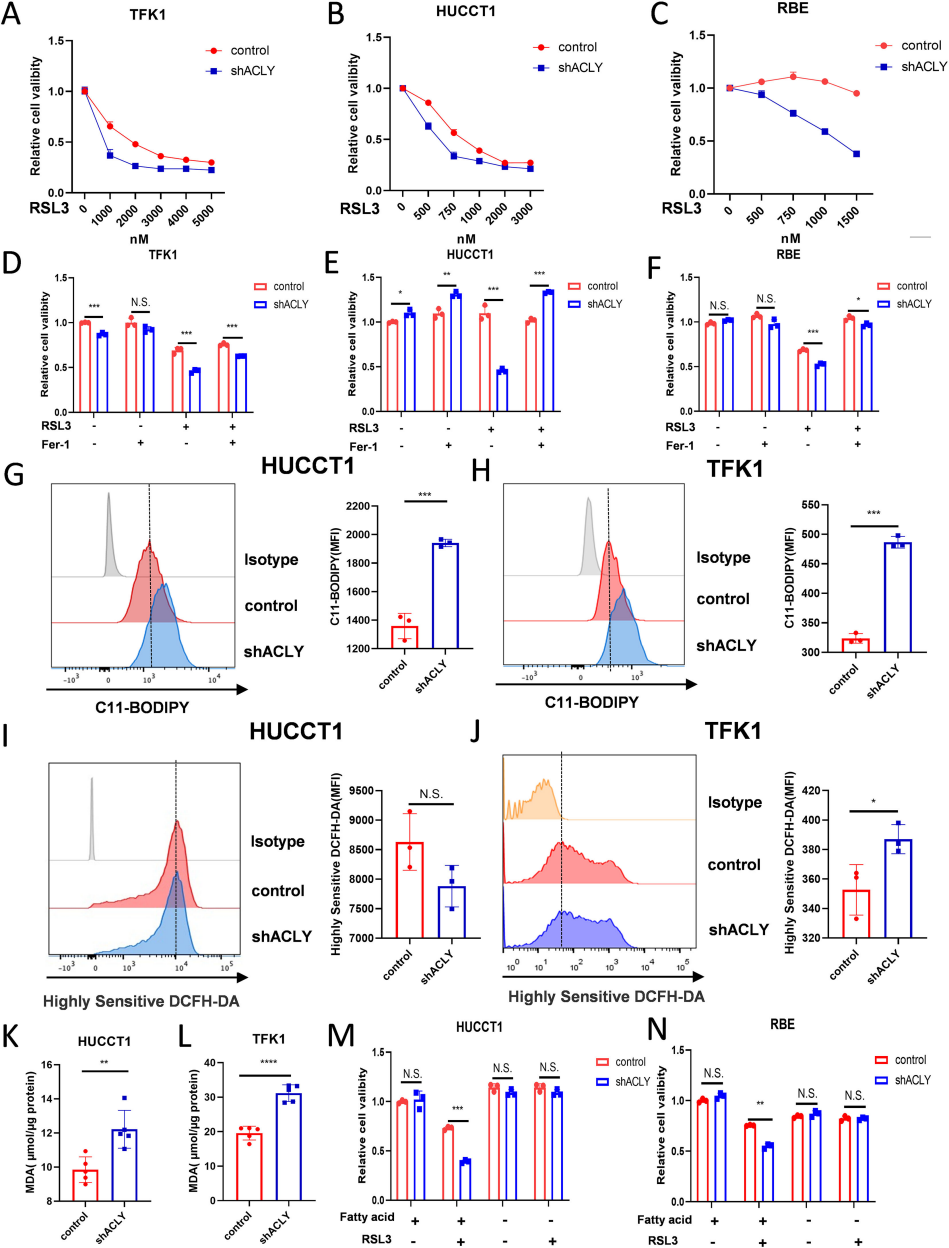
Inhibition of ACLY promotes ferroptosis in CCA cells. **(A-C)** CCK-8 assay of cell viability in control and ACLY-KD CCA cells after treatment with different concentrations of RSL3 for 24 h. **(D-F)** Morphological analysis of control and ACLY-KD CCA cells after treatment RSL3 for 24 h. **(D-F)** CCK-8 assay of cell viability in control and ACLY-KD CCA cells after treatment with RSL3(TFK1 3μM, HUCCT1 1μM and RBE 500nM) + Ferrostatin-1(1μM). **(G, H)** Lipid peroxidation levels in control and ACLY-KD cells were detected using C11-BODIPY staining in CCA cells. **(I, J)** Reactive oxygen species (ROS) levels in control and ACLY-KD cells were detected using Highly Sensitive DCFH-DA staining in CCA cells. **(K, L)** Quantification of malondialdehyde (MDA) contents by ELISA in control and ACLY-KD cells. **(M, N)** CCK-8 assay of cell viability in control and ACLY-KD CCA cells after treatment with RSL3 and either lipid-rich or delipidated fetal bovine serum (FBS). *P < 0.05, **P < 0.01 and ***P < 0.001; N.S., not significant.

Accumulating evidence implicates that PUFAs play a crucial role in ferroptosis. We aimed to determine whether ACLY affects ferroptosis in CCA cells. Cell viability assays showed that knockdown of ACLY increased the susceptibility of CCA cells to ferroptosis induced by RSL3 (**Figures 5A-C**). Additionally, we found that an ACLY inhibitor enhanced the susceptibility of CCA cells to RSL3 (**Supplementary Figures S4D-F**). To further confirm the mode of cell death promoted by the knockdown of ACLY in CCA cells under RSL3 treatment, we treated cells with the ferroptosis inhibitor ferrostatin-1 (Ferr-1). Results showed that Ferr-1 reversed ACLY-KD-induced cell death (**Figures 5D-F**). We next investigated intracellular levels of ROS and lipid peroxidation, which are two markers associated with ferroptosis. C11-BODIPY 581/591 staining and a malondialdehyde kit were employed to assess lipid peroxidation levels in both control and ACLY-KD cells, and a significant increase was observed in intracellular lipid peroxidation levels in ACLY-KD cells (**Figures 5G, H, K, L**). H2DCFDA staining was used to evaluate intracellular ROS levels, and the results indicated that ACLY-KD cells exhibited higher ROS levels in TFK1 cells (**Figures 5I, J**). To investigate the influence of exogenous fatty acids on RSL3-induced ferroptosis in ACLY-knockdown CCA cells, we cultured the cells in media supplemented with either lipid-enriched FBS or delipidated FBS. Results indicated that ACLY-knockdown cells did not exhibit increased sensitivity to the ferroptosis inducer in the medium supplemented with delipidated FBS (**Figures 5M, N**). In addition, we also found that ACLY knockdown resulted in elevated expression of the fatty acid transporter CD36, also known as fatty acid translocase, in HUCCT1 and TFK1 CCA cells (**Supplementary Figures S4G, H**). Taken together, these findings indicate that ACLY plays a crucial role as a regulator of ferroptosis in CCA, and that inhibition of ACLY promotes ferroptosis in CCA cells.

### 3.6 ACLY is associated with immune cell infiltration

**Figure 6:**
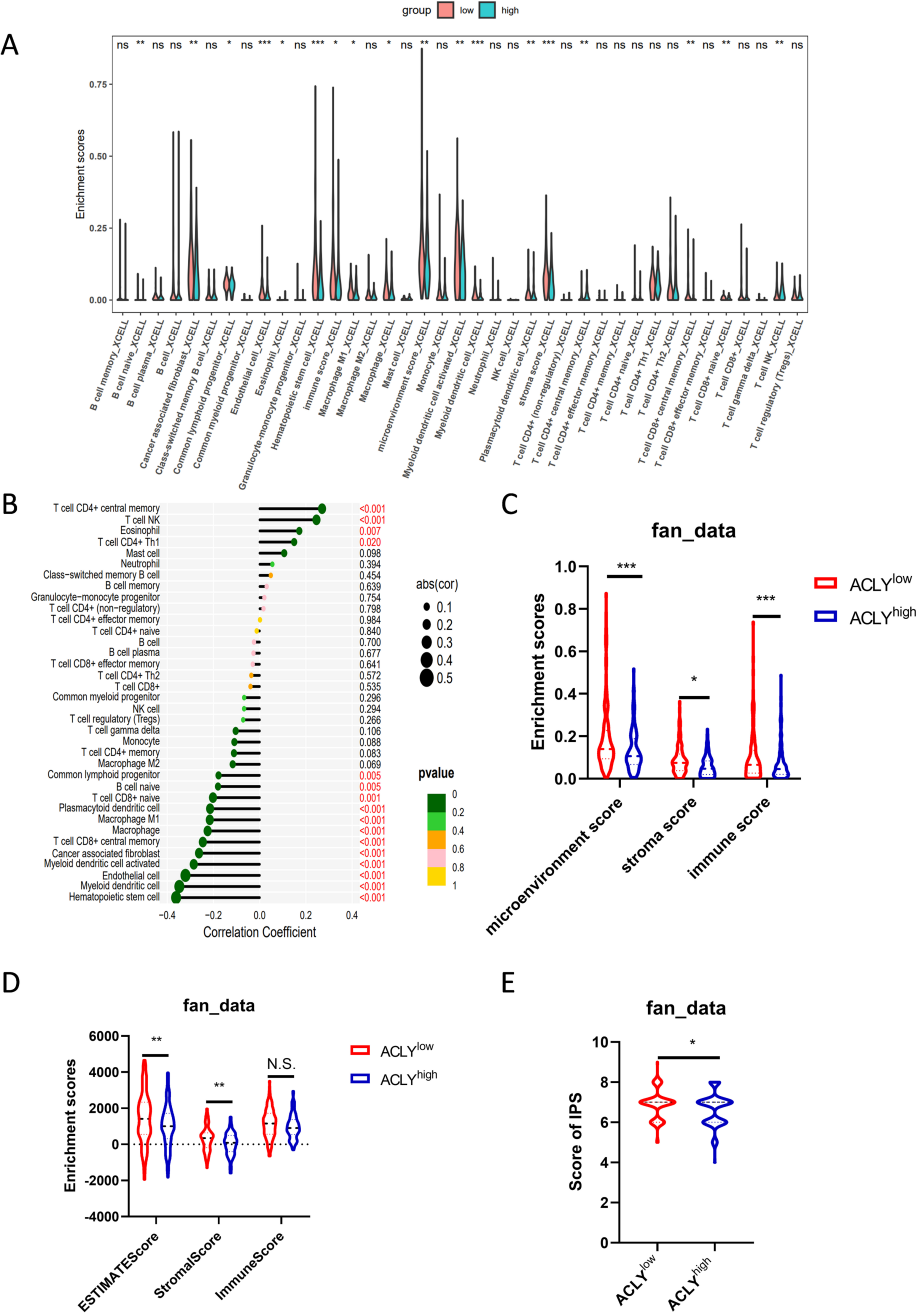
ACLY is associated with immune cell infiltration. **(A)** Grouped comparison of the infiltration levels of immune cells in the low and high expression groups of ACLY. **(B)** Results of the correlation between the expression of ACLY and immune cells. **(C)** Grouped comparison of the stromal score, immune score and microenviroment score, enriched with XCELL algorithm, in the low and high expression groups of ACLY. **(D)** Grouped comparison of the stromal score, immune score and ESTIMATE score, enriched with ESTIMATE algorithm, in the low and high expression groups of ACLY. **(E)** Grouped comparison of IPS (Immune cell Proportion Score) in the low and high expression groups of ACLY. *P < 0.05, **P < 0.01 and ***P < 0.001; N.S., not significant.

The role of ferroptosis in cancer immunity and immunotherapy has been a topic of substantial interest for years. To study the effect of ACLY expression on the tumor microenvironment, immune infiltration analysis was performed using the XCELL algorithm. The CCA cohort was divided into high and low expression groups based on ACLY expression levels to assess differences in immune cell infiltration between these groups. The result indicated that the levels of infiltration of central memory CD4^+^ T cells, natural killer T cells, and Th1 cells were significantly higher in the high ACLY expression group compared with the low expression group. Conversely, in hematopoietic stem cells, myeloid dendritic cells, endothelial cells, naïve CD8^+^ T cells, central memory CD8^+^ T cells, M1 macrophages, and cancer-associated fibroblasts, the level of infiltration was significantly higher in the low ACLY expression group compared with the high expression group (**Figure 6A**). Correlation analysis revealed that the expression of ACLY was positively correlated with central memory CD4^+^ T cells, natural killer T cells, and Th1 cells. In contrast, the expression of ACLY was negatively correlated with hematopoietic stem cells, myeloid dendritic cells, endothelial cells, naïve CD8^+^ T cells, central memory CD8^+^ T cells, M1 macrophages, and cancer-associated fibroblasts (**Figure 6B**). We also observed similar findings in other CCA cohort (**Supplementary Figures S5A, B**). In additional, the ESTIMATE algorithm indicated that CCA with higher ACLY expression had lower stromal scores and ESTIMATE scores (**Figure 6D**). Supporting this, the XCELL algorithm showed that the immune score, microenvironment score, and stromal score in the low expression group were significantly higher than those in the high expression group (**Figure 6C**). In another CCA cohorts, both of ESTIMATE algorithm and XCELL algorithm indicated that CCA with higher ACLY expression had lower immune score (**Supplementary Figures S5C, D**). Additionally, the results revealed that patients with higher ACLY expression had a lower Immune cell Proportion Score (IPS), suggesting that elevated ACLY expression is associated with a reduced benefit from immunotherapy (**Figure 6E**, **Supplementary Figure S5E**). In summary, these findings suggest that inhibition of ACLY could increase immune infiltration in CCA, highlighting its potential role in modulating the tumor microenvironment.

## 4 Discussion

Liver cancer is the fourth leading cause of cancer-related mortality worldwide (1, 23). Hepatocellular carcinoma (HCC) and cholangiocarcinoma (CCA) account for approximately 85% and 10% of all primary liver cancers, respectively (1, 23). Despite the extensive use of standard systemic therapies for advanced liver cancer, therapeutic resistance and disease progression remain prevalent (24). Precision medicine, a leading area in cancer research, has demonstrated significant success across various cancer types, although its application to liver cancer has been relatively limited (25). To advance personalized treatment strategies, researchers are investigating the molecular and microenvironmental features of liver cancer through various methodologies, including genomics, transcriptomics, proteomics, and spatial omics (22, 26, 27). Concurrently, targeted therapies and immunotherapies are being explored, though these approaches are still in their early stages (28). Given the current state of research, there is an urgent need to identify and validate novel molecular targets for liver cancer treatment. One such target is ATP citrate lyase (ACLY), a key enzyme in *de novo* lipogenesis (9). Aberrant expression of ACLY has been demonstrated in several tumors and is correlated with malignant tumor progression (2, 12, 15). In this study, we observed that ACLY expression in CCA tissues was significantly elevated compared to adjacent non-tumor tissues. Furthermore, higher ACLY expression was significantly associated with poorer prognosis. Subgroup analysis further revealed that elevated ACLY expression correlated with poor overall survival in CCA patients, particularly those with elder age, HBV infection, vascular invasion, and those undergoing adjuvant treatment. These findings suggest that patients with these characteristics might benefit the most from ACLY-targeted therapy. In summary, we thought that ACLY was a promising therapy target.

To elucidate the potential mechanisms of ACLY regulation, we constructed a protein-protein interaction (PPI) network using differentially expressed genes between the ACLY low and high expression groups. This analysis identified several hub genes, including APOH, APOB, APOA1, and APOA2, which play significant roles in cancer progression. Apolipoproteins have been reported to be crucial in the progression of various cancers and hold promise for diagnostic and prognostic applications (29). For example, APOH is notably downregulated in hepatocellular carcinoma, suggesting its potential as a tumor biomarker (30). Similarly, reduced plasma levels of APOA1 are associated with metastasis and poor prognosis across several cancers, such as ovarian, colorectal, and esophageal cancers. Both *in vitro* and *in vivo* studies have demonstrated that APOA1 can inhibit tumor initiation and progression (31–34). Additionally, mutations leading to APOB inactivation are correlated with the overexpression of oncogenic regulators and the downregulation of tumor suppressors, resulting in poorer survival outcomes in hepatocellular carcinoma (35). These findings indicate that apolipoproteins are involved in the malignant progression of CCA and are regulated by ACLY. To further explore this, we conducted Gene Ontology (GO) and KEGG pathway analyses. Our results revealed that upregulated differentially expressed genes (DEGs) are enriched in pathways related to the cell cycle, focal adhesion, p53 signaling, and mTOR signaling. In contrast, downregulated DEGs were associated with fatty acid metabolism, amino acid metabolism, and other related pathways. Gene Set Enrichment Analysis (GSEA) further showed that high ACLY expression is positively correlated with epithelial-mesenchymal transition and angiogenesis, suggesting that ACLY may influence metastasis-related pathways. Conversely, high ACLY expression was negatively correlated with the G2M checkpoint, MYC targets, and E2F targets, indicating an inverse relationship with cancer proliferation and anti-tumor immunity. These observations align with previous studies and highlight the multifaceted role of ACLY in cancer progression.

The mechanisms by which ACLY induces tumor progression are not fully understood. It was reported that ACLY promoted the epithelial-mesenchymal transition (EMT) and cancer stemness of colorectal cancer and hepatocellular carcinoma cells through the Wnt/β-catenin pathway (15, 36, 37). In this study, we found that high ACLY expression was positively correlated with epithelial-mesenchymal transition. In addition, stemness analysis indicated that ACLY exhibited a strong positive correlation with the GeneCardseveral stemness signature, and certain higher GeneCard stemness score was associated with poor prognosis. These results indicate that tumor-initiating cells play a crucial role in the malignant progression of CCA, and ACLY serves as a key modulator in maintaining CCA stemness. Additionally, ACLY’s nuclear translocation has been shown to enhance proliferation of endometrial cancer cells by regulating pyrimidine metabolism (38). Furthermore, nuclear acetyl-CoA production by ACLY facilitates homologous recombination and induces tumor cell resistance to DNA-damaging drugs (39). In the context of cancer immunotherapy, ACLY inhibition has been reported to overcome immunotherapy resistance through polyunsaturated fatty acid peroxidation and activation of the cGAS-STING pathway (2). As key rate-limiting enzyme in *de novo* fatty acid synthesis, silencing ACLY can inhibit the proliferation of multiple tumor cells lines, and exogenous supplementation with fatty acids or cholesterol can partially counteract the proliferation inhibition effect (40, 41). Despite these insights, the function and underlying mechanisms of ACLY in CCA have yet to be explored.

In this study, we observed that ACLY inhibition did not impact the proliferation or migration of RBE and HUCCT1 CCA cells, whereas it inhibited these processes in TFK1 cells. This discrepancy may be attributed to the high heterogeneity among cholangiocarcinoma cells, which exhibit varying gene mutation patterns (42, 43). Given ACLY’s role as a key enzyme in *de novo* fatty acid synthesis, its inhibition results in reduced synthesis of intracellular saturated fatty acids (44). What’s more, function analysis of ACLY-related differentially expressed genes revealed that ACLY was associated with apolipoproteins, unsaturated fatty acid synthesis, and linoleic acid metabolism. In additionally, in our previous study, we found that inhibition of ACLY disrupts the balance between saturated and unsaturated fatty acids within tumor cells (2). Thus, we concluded that ACLY plays a crucial role in maintaining fatty acid homeostasis. Consequently, we assessed cell proliferation when treated with different fatty acids. We found that polyunsaturated fatty acids significantly inhibited the proliferation of ACLY-knockdown tumor cells. Additionally, PUFAs are known to induce ferroptosis, a form of regulated cell death (18). Our findings also demonstrated that ACLY knockdown or pharmacological inhibition promotes ferroptosis in tumor cells, as evidenced by a significant increase in intracellular lipid peroxides and malondialdehyde (MDA) levels. Based on these results, we propose that inhibiting ACLY can enhance ferroptosis in tumor cells, highlighting its potential as a therapeutic target.

Abnormal lipid metabolism is a hallmark of tumor cells, which rely on both *de novo* fatty acid synthesis and exogenous uptake to meet their lipid requirements. Compared to normal cells, tumor cells preferentially engage in *de novo* fatty acid synthesis to mitigate the risk associated with excessive intake of unsaturated fatty acids (45, 46). Polyunsaturated fatty acids (PUFAs) are particularly susceptible to lipid peroxidation, which increases tumor cell sensitivity to oxidative stress-induced cell death (47, 48). In our study, we observed that inhibiting ACLY led to suppression of the *de novo* fatty acid synthesis pathway. Concurrently, ACLY knockdown resulted in elevated expression of the fatty acid transporter CD36, also known as fatty acid translocase. This increase in CD36 expression facilitated enhanced uptake of unsaturated fatty acids by the tumor cells. The accumulated PUFAs then underwent lipid peroxidation under oxidative stress conditions, leading to ferroptosis in the tumor cells. Additionally, when cultured in lipid-depleted medium, ACLY-knockdown cells did not exhibit increased sensitivity to ferroptosis inducers. This finding suggests that the enhanced uptake of unsaturated fatty acids due to ACLY inhibition is crucial for promoting ferroptosis in tumor cells. Therefore, we propose that inhibiting ACLY promotes ferroptosis by increasing the tumor cells’ uptake of PUFAs, which are then subject to lipid peroxidation under oxidative stress.

The role of ferroptosis in cancer immunity and immunotherapy has attracted substantial interest in recent years. Ferroptosis has been shown to be involved in the anti-tumor effects of CD8^+^ T cells and enhance the efficacy of anti-PD-1/PD-L1 immunotherapy. Combining immunotherapy with ferroptosis-inducing treatments, such as radiotherapy and targeted therapy, produces synergistic effects in tumor control (49–51). In additionally, our previous study demonstrated that ACLY inhibition overcomes immunotherapy resistance through polyunsaturated fatty acid peroxidation and activation of the cGAS-STING pathway (2). In the current study, we observed a negative correlation between ACLY expression and central memory CD8+ T cells, immune score, and IPS score. These findings suggest that ACLY inhibition may enhance the efficacy of immunotherapy in cholangiocarcinoma (CCA). Thus, combining ACLY inhibition with immunotherapy represents a promising therapeutic strategy for CCA. Furthermore, we discovered that pharmacological inhibition of ACLY synergizes with ferroptosis inducers and increases the sensitivity of CCA cells to PUFAs. Notably, bempedoic acid, a specific ACLY inhibitor, has recently been approved by the U.S. Food and Drug Administration (FDA) for lowering low-density lipoprotein cholesterol. We had found that BemA combined with αPD-L1 treatment exhibit strong anti-tumor effect. In conclusion, we propose that patients with high ACLY expression might benefit from pharmacological inhibition of ACLY in combination with immunotherapy, PUFAs, or ferroptosis-inducing treatments.

In recent years, mounting evidence suggests that ACLY is involved in the malignant progression of tumors. However, the function and underlying mechanisms of ACLY in CCA remain largely unexplored and poorly understood. This study is the first to demonstrate that ACLY expression is upregulated in CCA and is inversely correlated with prognosis. In addition, we constructed a clinical prediction model that demonstrates superior predictive capability. Mechanistically, we found that inhibition of ACLY promotes ferroptosis in CCA cells, a phenomenon that has not been previously reported. Additionally, our analyses revealed that ACLY expression levels are negatively correlated with cancer stemness, immune infiltration, and the efficacy of immunotherapy in CCA. Finally, we propose a potential therapeutic strategy that combines ACLY inhibitors with ferroptosis inducers for the treatment of CCA. However, the study primarily focuses on the biological behavior of ACLY inhibition in CCA cells, and the precise mechanisms warrant further investigation.

## Data Availability

The datasets presented in this study can be found in online repositories. The names of the repository/repositories and accession number(s) can be found in the article/**Supplementary Material**.

## Supplementary material

Supplementary Figure 1 Kaplan–Meier survival analysis of ACLY expression and OS in different patient subgroups with distinct clinicopathological factors.

Supplementary Figure 2 Functional enrichment analysis of ACLY in cholangiocarcinoma. **(A)** Volcano plot of single gene differential analysis of ACLY. **(B)** Protein–protein interaction network (PPI) of differentially expressed genes in single gene differential analysis. **(C)** Protein–protein interaction network map (PPI) of the HUB gene. **(D)** Single gene co-expression heatmap of the HUB gene and PPP1R14B. **(E, G)** Results of the correlation between the expression of ACLY and stemness scores in GSE244807 and E-MTAB-6389 cohort. **(F, H)** Forest plot showing correlation of different stemness signatures with overall survival in GSE244807 and E-MTAB-6389 cohort.

Supplementary Figure 3 Inhibition of ACLY has no significant effect on the migration capability of cholangiocarcinoma (CCA) cells. **(A)** The protein expression of ACLY in 4 CHOL cell lines (RBE, HUCCT1, 9810 and TFK1) was measured by Western blot. **(B-D)** The migratory capability of CCA cells was assessed using a transwell assay.

Supplementary Figure 4 Inhibition of ACLY promotes ferroptosis in cholangiocarcinoma cells by increasing the uptake of polyunsaturated fatty acids (PUFAs). **(A-C)** Morphological analysis of control and ACLY-KD CCA cells after treatment RSL3 for 24 h. **(D-F)** CCK-8 assay of cell viability in control and ACLY-KD CCA cells after treatment with RSL3 (TFK1 2μM, HUCCT1 1μM, RBE 500nM) and SB-204900 (20μM) for 24h. **(G, H)** Immunoblot analysis of CD36 expression in control and ACLY KD cells.

Supplementary Figure 5 ACLY is associated with immune cell infiltration. **(A)** Grouped comparison of immune cell infiltration levels between low and high ACLY expression groups in the GSE89749 cohort. **(B)** Results of the correlation between the expression of ACLY and immune cells in GSE89749 cohort. **(C)** Grouped comparison of stromal score, immune score, and microenvironment score, as assessed using the XCELL algorithm, between low and high ACLY expression groups in the GSE89749 cohort. **(D)** Grouped comparison of stromal score, immune score, and ESTIMATE score, as assessed using the ESTIMATE algorithm, between low and high ACLY expression groups. **(E)** Grouped comparison of IPS (Immune cell Proportion Score) between the low and high expression groups of ACLY in the GSE89749 cohort.

## References

[1] Brindley PJ Bachini M Ilyas SI Khan SA Loukas A Sirica AE. Cholangiocarcinoma. Nat Rev Dis Primers. (2021) 7:65. doi: 10.1038/s41572-021-00300-2 34504109 PMC9246479

[2] Xiang W Lv H Xing F Sun X Ma Y Wu L. Inhibition of ACLY overcomes cancer immunotherapy resistance via polyu nsaturated fatty acids peroxidation and cGAS-STING activation. Sci Adv. (2023) 9:eadi2465. doi: 10.1126/sciadv.adi2465 38055816 PMC10699784

[3] Shroff RT Kennedy EB Bachini M Bekaii-Saab T Crane C Edeline J. Adjuvant therapy for resected biliary tract cancer: ASCO clinical practice guideline. J Clin Oncol. (2019) 37:1015–27. doi: 10.1200/jco.18.02178 30856044

[4] Valle JW Lamarca A Goyal L Barriuso J Zhu AX. New horizons for precision medicine in biliary tract cancers. Cancer Discovery. (2017) 7:943–62. doi: 10.1158/2159-8290.Cd-17-0245 PMC5586506 28818953

[5] Valle JW. Advances in the treatment of metastatic or unresectable biliary tract cancer. Ann Oncol. (2010) 21 Suppl 7:vii345–8. doi: 10.1093/annonc/mdq420 20943640

[6] Hanahan D. Hallmarks of cancer: new dimensions. Cancer Discovery. (2022) 12:31–46. doi: 10.1158/2159-8290.Cd-21-1059 35022204

[7] Menendez JA Lupu R. Fatty acid synthase and the lipogenic phenotype in cancer pathogenesis. Nat Rev Cancer. (2007) 7:763–77. doi: 10.1038/nrc2222 17882277

[8] Martin-Perez M Urdiroz-Urricelqui U Bigas C Benitah SA. The role of lipids in cancer progression and metastasis. Cell Metab. (2022) 34:1675–99. doi: 10.1016/j.cmet.2022.09.023 36261043

[9] Granchi C. ATP citrate lyase (ACLY) inhibitors: An anti-cancer strategy at the crossroads of glucose and lipid metabolism. Eur J Med Chem. (2018) 157:1276–91. doi: 10.1016/j.ejmech.2018.09.001 30195238

[10] Guertin DA Wellen KE. Acetyl-CoA metabolism in cancer. Nat Rev Cancer. (2023) 23:156–72. doi: 10.1038/s41568-022-00543-5 PMC11137663 36658431

[11] Qian X Hu J Zhao J Chen H. ATP citrate lyase expression is associated with advanced stage and prognosis in gastric adenocarcinoma. Int J Clin Exp Med. (2015) 8:7855–60. PMC4509285 26221340

[12] Gao Y Islam MS Tian J Lui VW Xiao D. Inactivation of ATP citrate lyase by Cucurbitacin B: A bioactive compound from cucumber, inhibits prostate cancer growth. Cancer Lett. (2014) 349:15–25. doi: 10.1016/j.canlet.2014.03.015 24690568

[13] Li J Condello S Thomes-Pepin J Ma X Xia Y Hurley TD. Lipid desaturation is a metabolic marker and therapeutic target of ovarian cancer stem cells. Cell Stem Cell. (2017) 20:303–14.e5. doi: 10.1016/j.stem.2016.11.004 28041894 PMC5337165

[14] Migita T Narita T Nomura K Miyagi E Inazuka F Matsuura M. ATP citrate lyase: activation and therapeutic implications in non-small cell lung cancer. Cancer Res. (2008) 68:8547–54. doi: 10.1158/0008-5472.Can-08-1235 18922930

[15] Han Q Chen C-A Yang W Liang D Lv H-W Lv G-S. ATP-citrate lyase regulates stemness and metastasis in hepatocellular carcinoma via the Wnt/β-catenin signaling pathway. Hepatobil pancreatic Dis international: HBPD Int. (2021) 20:251–61. doi: 10.1016/j.hbpd.2020.05.010 33129711

[16] Adorno-Cruz V Hoffmann AD Liu X Dashzeveg NK Taftaf R Wray B. ITGA2 promotes expression of ACLY and CCND1 in enhancing breast cancer stemness and metastasis. Genes Dis. (2021) 8:493–508. doi: 10.1016/j.gendis.2020.01.015 34179312 PMC8209312

[17] Jiang X Stockwell BR Conrad M. Ferroptosis: mechanisms, biology and role in disease. Nat Rev Mol Cell Biol. (2021) 22:266–82. doi: 10.1038/s41580-020-00324-8 PMC8142022 33495651

[18] Dierge E Debock E Guilbaud C Corbet C Mignolet E Mignard L. Peroxidation of n-3 and n-6 polyunsaturated fatty acids in the acidic tumor environment leads to ferroptosis-mediated anticancer effects. Cell Metab. (2021) 33:1701–15.e5. doi: 10.1016/j.cmet.2021.05.016 34118189

[19] Qiu B Zandkarimi F Bezjian CT Reznik E Soni RK Gu W. Phospholipids with two polyunsaturated fatty acyl tails promote ferroptosis. Cell. (2024) 187:1177–90.e18. doi: 10.1016/j.cell.2024.01.030 38366593 PMC10940216

[20] Bartolacci C Andreani C Vale G Berto S Melegari M Crouch AC. Targeting *de novo* lipogenesis and the Lands cycle induces ferroptosis in KRAS-mutant lung cancer. Nat Commun. (2022) 13:4327. doi: 10.1038/s41467-022-31963-4 35882862 PMC9325712

[21] Dufort FJ Gumina MR Ta NL Tao Y Heyse SA Scott DA. Glucose-dependent *de novo* lipogenesis in B lymphocytes: a requirement for atp-citrate lyase in lipopolysaccharide-induced differentiation. J Biol Chem. (2014) 289:7011–24. doi: 10.1074/jbc.M114.551051 PMC3945362 24469453

[22] Dong L Lu D Chen R Lin Y Zhu H Zhang Z. Proteogenomic characterization identifies clinically relevant subgroups of intrahepatic cholangiocarcinoma. Cancer Cell. (2022) 40:70–87.e15. doi: 10.1016/j.ccell.2021.12.006 34971568

[23] Llovet JM Zucman-Rossi J Pikarsky E Sangro B Schwartz M Sherman M. Hepatocellular carcinoma. Nat Rev Dis Primers. (2016) 2:16018. doi: 10.1038/nrdp.2016.18 27158749

[24] Banales JM Marin JJG Lamarca A Rodrigues PM Khan SA Roberts LR. Cholangiocarcinoma 2020: the next horizon in mechanisms and management. Nat Rev Gastroenterol Hepatol. (2020) 17:557–88. doi: 10.1038/s41575-020-0310-z PMC7447603 32606456

[25] Yang X Yang C Zhang S Geng H Zhu AX Bernards R. Precision treatment in advanced hepatocellular carcinoma. Cancer Cell. (2024) 42:180–97. doi: 10.1016/j.ccell.2024.01.007 38350421

[26] Yang C Zhang S Cheng Z Liu Z Zhang L Jiang K. Multi-region sequencing with spatial information enables accurate heterogeneity estimation and risk stratification in liver cancer. Genome Med. (2022) 14:142. doi: 10.1186/s13073-022-01143-6 36527145 PMC9758830

[27] Gao Q Zhu H Dong L Shi W Chen R Song Z. Integrated proteogenomic characterization of HBV-related hepatocellular carcinoma. Cell. (2019) 179:561–77.e22. doi: 10.1016/j.cell.2019.08.052 31585088

[28] Li Y Yang C Liu Z Du S Can S Zhang H. Integrative analysis of CRISPR screening data uncovers new opportunities for optimizing cancer immunotherapy. Mol Cancer. (2022) 21:2. doi: 10.1186/s12943-021-01462-z 34980132 PMC8722047

[29] Ren L Yi J Li W Zheng X Liu J Wang J. Apolipoproteins and cancer. Cancer Med. (2019) 8:7032–43. doi: 10.1002/cam4.2587 PMC6853823 31573738

[30] Liu Y Wu Z Zhao Y Zhen M Wang Y Liu Q. Apolipoprotein H-based prognostic risk correlates with liver lipid met abolism disorder in patients with HBV-related hepatocellular carcinoma. Heliyon. (2024) 10:e31412. doi: 10.1016/j.heliyon.2024.e31412 38831828 PMC11145473

[31] Marinho AT Lu H Pereira SA Monteiro E Gabra H Recchi C. Anti-tumorigenic and platinum-sensitizing effects of apolipoprotein A1 and apolipoprotein A1 mimetic peptides in ovarian cancer. Front Pharmacol. (2018) 9:1524. doi: 10.3389/fphar.2018.01524 30745873 PMC6360149

[32] Zhang X Zhao X-W Liu D-B Han C-Z Du L-L Jing J-X. Lipid levels in serum and cancerous tissues of colorectal cancer patients. World J Gastroenterol. (2014) 20:8646–52. doi: 10.3748/wjg.v20.i26.8646 PMC4093716 25024621

[33] Pan Y Ye Z Ling Y Kong L Wang C Chen G. The apolipoprotein B and apolipoprotein A-I Ratio serves as a strong p rognostic factor for the overall survival of patients with colorectal cancer. Front Oncol. (2023) 12:1089688. doi: 10.3389/fonc.2022.1089688 36713523 PMC9880464

[34] Wang X-P Li X-H Zhang L Lin J-H Huang H Kang T. High level of serum apolipoprotein A-I is a favorable prognostic factor for overall survival in esophageal squamous cell carcinoma. BMC Cancer. (2016) 16:516. doi: 10.1186/s12885-016-2502-z 27444612 PMC4957343

[35] Lee G Jeong YS Kim DW Kwak MJ Koh J Joo EW. Clinical significance of APOB inactivation in hepatocellular carcinoma. Exp Mol Med. (2018) 50:1–12. doi: 10.1038/s12276-018-0174-2 PMC6235894 30429453

[36] Wen J Min X Shen M Hua Q Han Y Zhao L. ACLY facilitates colon cancer cell metastasis by CTNNB1. J Exp Clin Cancer research: CR. (2019) 38:401. doi: 10.1186/s13046-019-1391-9 31511060 PMC6740040

[37] Hu C Xin Z Sun X Hu Y Zhang C Yan R. Activation of ACLY by SEC63 deploys metabolic reprogramming to facilitate hepatocellular carcinoma metastasis upon endoplasmic reticulum stress. J Exp Clin Cancer research: CR. (2023) 42:108. doi: 10.1186/s13046-023-02656-7 37122003 PMC10150531

[38] Dai M Yang B Chen J Liu F Zhou Y Zhou Y. Nuclear-translocation of ACLY induced by obesity-related factors enhances pyrimidine metabolism through regulating histone acetylation in endometrial cancer. Cancer Lett. (2021) 513:36–49. doi: 10.1016/j.canlet.2021.04.024 33991616

[39] Sivanand S Rhoades S Jiang Q Lee JV Benci J Zhang J. Nuclear acetyl-coA production by ACLY promotes homologous recombination. Mol Cell. (2017) 67:252–65.e6. doi: 10.1016/j.molcel.2017.06.008 28689661 PMC5580398

[40] Zaidi N Royaux I Swinnen JV Smans K. ATP citrate lyase knockdown induces growth arrest and apoptosis through different cell- and environment-dependent mechanisms. Mol Cancer Ther. (2012) 11:1925–35. doi: 10.1158/1535-7163.Mct-12-0095 22718913

[41] Lin R Tao R Gao X Li T Zhou X Guan KL. Acetylation stabilizes ATP-citratelyase to promote lipid biosynthesis and tumor growth. Mol Cell. (2013) 51:506–18. doi: 10.1016/j.molcel.2013.07.002 PMC4180208 23932781

[42] Wang XY Zhu WW Wang Z Huang JB Wang SH Bai FM. Driver mutations of intrahepatic cholangiocarcinoma shape clinically relevant genomic clusters with distinct molecular features and therapeutic vulnerabilities. Theranostics. (2022) 12:260–76. doi: 10.7150/thno.63417 PMC8690927 34987644

[43] Liu D Shi Y Chen H Nisar MA Jabara N Langwinski N. Molecular profiling reveals potential targets in cholangiocarcinoma. World J Gastroenterol. (2023) 29:4053–71. doi: 10.3748/wjg.v29.i25.4053 PMC10354586 37476584

[44] Roumans KHM Lindeboom L Veeraiah P Remie CME Phielix E Havekes B. Hepatic saturated fatty acid fraction is associated with *de novo* lipogenesis and hepatic insulin resistance. Nat Commun. (2020) 11:1891. doi: 10.1038/s41467-020-15684-0 32312974 PMC7170906

[45] Medes G Thomas A Weinhouse S. Metabolism of neoplastic tissue. IV. A study of lipid synthesis in neoplastic tissue slices in *vitro* . Cancer Res. (1953) 13:27–9. 13032945

[46] Currie E Schulze A Zechner R Walther TC Farese RV Jr. Cellular fatty acid metabolism and cancer. Cell Metab. (2013) 18:153–61. doi: 10.1016/j.cmet.2013.05.017 PMC3742569 23791484

[47] Rysman E Brusselmans K Scheys K Timmermans L Derua R Munck S. *De novo* lipogenesis protects cancer cells from free radicals and chemotherapeutics by promoting membrane lipid saturation. Cancer Res. (2010) 70:8117–26. doi: 10.1158/0008-5472.Can-09-3871 20876798

[48] Yin H Xu L Porter NA. Free radical lipid peroxidation: mechanisms and analysis. Chem Rev. (2011) 111:5944–72. doi: 10.1021/cr200084z 21861450

[49] Lang X Green MD Wang W Yu J Choi JE Jiang L. Radiotherapy and immunotherapy promote tumoral lipid oxidation and ferroptosis via synergistic repression of SLC7A11. Cancer Discovery. (2019) 9:1673–85. doi: 10.1158/2159-8290.Cd-19-0338 PMC6891128 31554642

[50] Wang W Green M Choi JE Gijón M Kennedy PD Johnson JK. CD8(+) T cells regulate tumour ferroptosis during cancer immunotherapy. Nature. (2019) 569:270–4. doi: 10.1038/s41586-019-1170-y PMC6533917 31043744

[51] Jiang Z Lim SO Yan M Hsu JL Yao J Wei Y. TYRO3 induces anti-PD-1/PD-L1 therapy resistance by limiting innate immunity and tumoral ferroptosis. J Clin Invest. (2021) 131:e139434. doi: 10.1172/jci139434 33855973 PMC8262501

