# Supplementary figures and images for: The pathological significance and potential mechanism of ACLY in cholangiocarcinoma
Source: Front Immunol. 2024 Sep 27;15:1477267. doi: 10.3389/fimmu.2024.1477267 (PMC11466796; doi:10.3389/fimmu.2024.1477267)

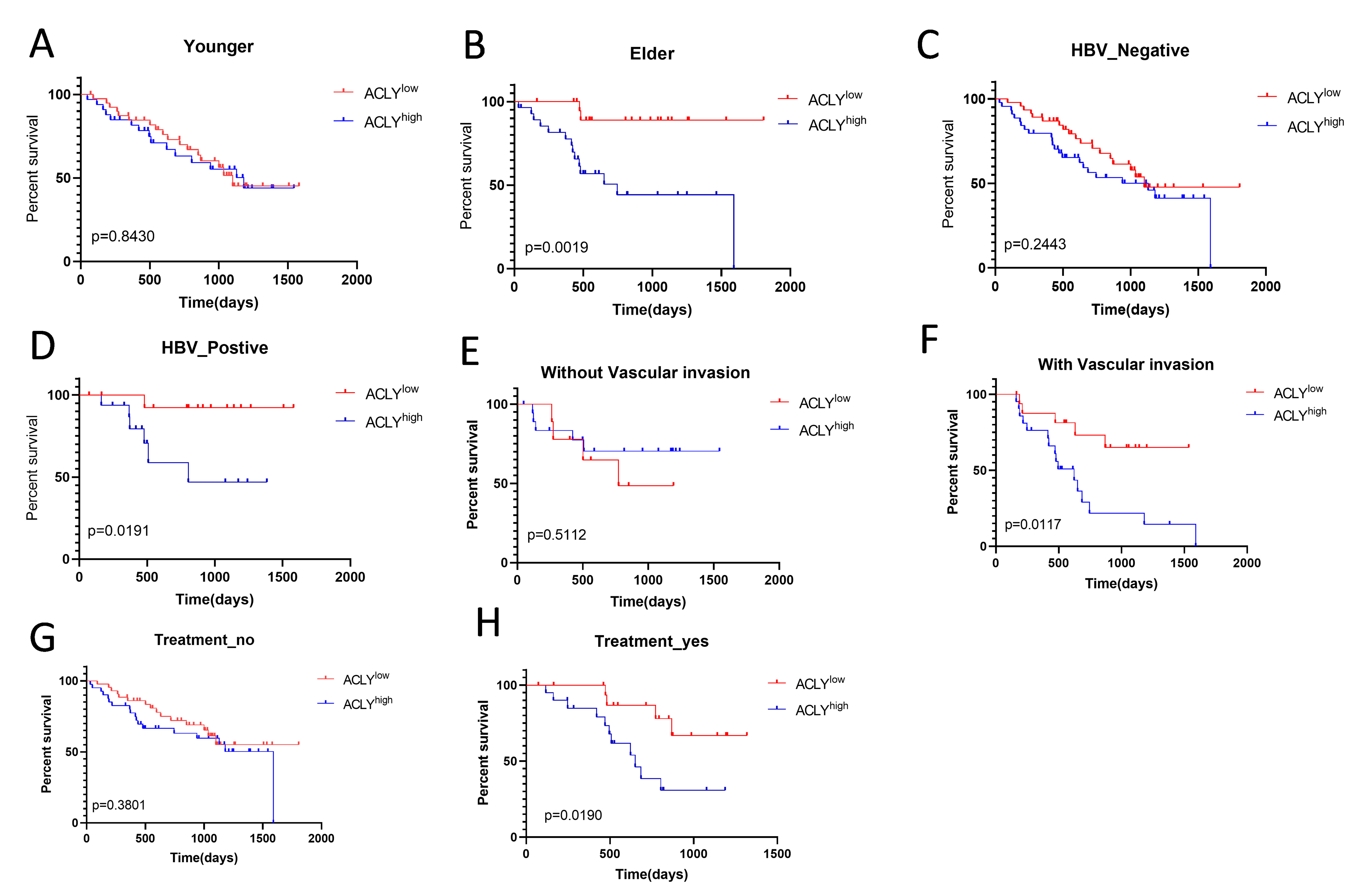

Supplement: Supplementary Figure 1 — Kaplan–Meier survival analysis of ACLY expression and OS in different patient subgroups with distinct clinicopathological factors. [file Image1.jpeg]

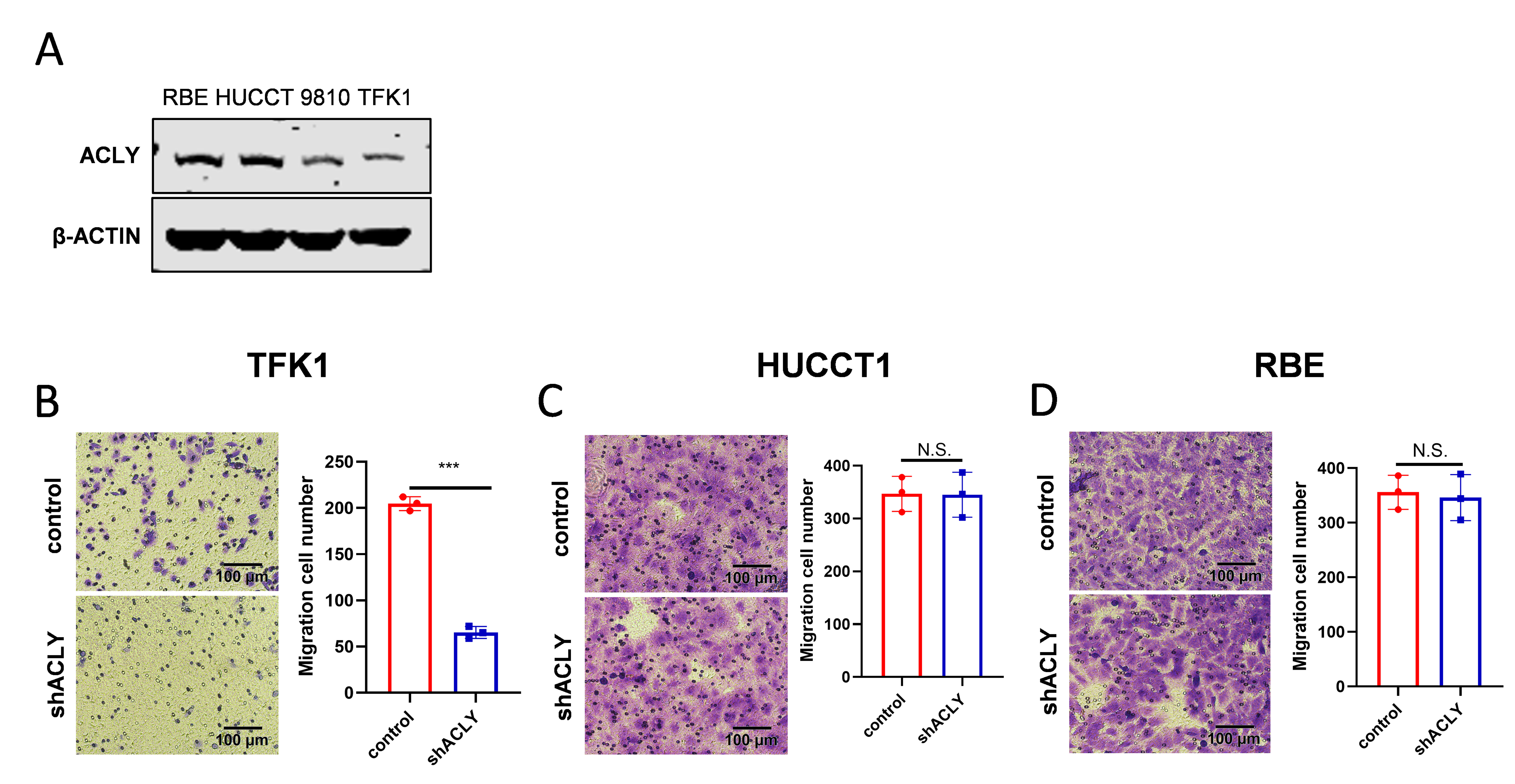

Supplement: Supplementary Figure 3 — Inhibition of ACLY has no significant effect on the migration capability of cholangiocarcinoma (CCA) cells. (A) The protein expression of ACLY in 4 CHOL cell lines (RBE, HUCCT1, 9810 and TFK1) was measured by Western blot. (B-D) The migratory capability of CCA cells was assessed using a transwell assay. [file Image3.jpeg]
